# BUNDLE OF MEASURES TO SUPPORT INTRAHOSPITAL EXCLUSIVE BREASTFEEDING: EVIDENCE OF SYSTEMATIC REVIEWS

**DOI:** 10.1590/1984-0462/;2018;36;2;00002

**Published:** 2018-04-23

**Authors:** Kelly Pereira Coca, Vânia Lopes Pinto, Flavia Westphal, Pâmilla Nayara Alves Mania, Ana Cristina Freitas de Vilhena Abrão

**Affiliations:** a Universidade Federal de São Paulo, São Paulo, SP, Brasil.

**Keywords:** Breast feeding, Weaning, Rooming-in care, Risk factors, Aleitamento materno, Desmame, Alojamento conjunto, Fatores de risco

## Abstract

**Objective:**

To identify the main recommendations found in systematic reviews regarding exclusive breastfeeding protective factors.

**Data source:**

Integrative review based on the guiding question: What evidence is found in literature regarding the protective factors of exclusive breastfeeding during the intrahospital period? A search was conducted in the Cochrane Library, PubMed/MEDLINE and LILACS database using the keyword “Breast Feeding” and the word “Breastfeeding”. Systematic reviews published from 2007 to 2016 that answered the guiding question were included in the study, whereas systematic reviews that analyzed breastfeeding of preterm infants and breastfeeding of children with orofacial malformation were excluded. The sample included eight systematic reviews.

**Data synthesis:**

The recommendations related to the protective factors for exclusive in-hospital breastfeeding found in the systematic reviews were: early skin-to-skin contact, rooming-in care, intervention for treating painful nipples during breastfeeding, restriction of infant supplementation, baby-led breastfeeding and educational interventions and support for mothers during hospital stay. The proposed measures included the six practices presented as protective factors.

**Conclusions:**

The review enabled the identification of evidence to support the recommended measures from delivery room to hospital discharge, with the aim of encouraging breastfeeding and preventing intrahospital weaning,

## RESUMO

**Objetivo:**

Identificar as principais recomendações encontradas em revisões sistemáticas relacionadas aos fatores de proteção do aleitamento materno exclusivo intra-hospitalar.

**Fonte de dados:**

Revisão integrativa partindo da questão norteadora: Quais são as evidências identificadas na literatura relacionadas aos fatores de proteção ao aleitamento materno exclusivo no período intra-hospitalar? Foi realizada uma busca nas bases de dados Cochrane Library, PubMed/MEDLINE e LILACS por meio do descritor *Breast Feeding* e da palavra *Breastfeeding*. Os critérios de inclusão foram revisões sistemáticas publicadas entre 2007 e 2016 que respondiam à questão norteadora; e os critérios de exclusão, revisões sistemáticas que analisaram o aleitamento de crianças prematuras e com má formação orofacial. A amostra foi composta por oito revisões sistemáticas.

**Síntese dos dados:**

As recomendações relacionadas aos fatores de proteção ao aleitamento materno exclusivo intra-hospitalar encontradas nas revisões sistemáticas foram: o contato pele a pele precoce; a permanência da criança em alojamento conjunto; a intervenção na dor mamilar durante a amamentação; a restrição do uso de suplementação para lactentes; o aleitamento materno sob livre demanda; e as intervenções educativas por meio de suporte individual e/ou em grupos durante a internação. O conjunto de medidas proposto incluiu todas as seis práticas apresentadas.

**Conclusões:**

A revisão possibilitou a identificação de evidências que embasaram as medidas propostas com a finalidade de incentivar o aleitamento materno exclusivo intra-hospitalar, iniciado na sala de parto e seguido pela internação até a alta hospitalar.

## INTRODUCTION

The superior quality of human milk in relation to other forms of feeding is acknowledged, and exclusive breastfeeding (EBF) is the best food for the children.^1^ Such a practice represents a significant impact on public health in the world, capable of preventing the death of 823 thousand children aged less than 5 years and 20 thousand women every year, besides saving 300 billion dollars, according to estimations made in 2016.^2^

Despite the recommendations and the benefits of breastfeeding, it is very difficult to increase the rates of EBF.^3^ ^,^ ^4^ The latest prevalence study in Brazil showed that 87.3% of the children with 30 days of life were being breastfed (BF), and, of these, only 47.5% were in EBF. Children with 180 days of life presented lower rates of BF, 68.6%, whereas the exclusivity was present in only 7.7% of them.^5^

Special situations such as prematurity, health conditions that separate the mother from the newborn, introduction of artificial formula, hospital routine that limits the practice of BF, use of medications that contraindicate breastfeeding, presence of pain and/or nipple damage, negative maternal experience in the previous breastfeeding, mother returning to work before the six months of life of the child, maternal insecurity, myths from society, lack of family support and lack of preparation from health professionals are also related to early weaning.^3^ ^,^ ^4^ The lack of preparation of the woman regarding breastfeeding is also a factor that influences the reduction of BF.^6^ Other limiting factors to the stimulation of breastfeeding are maternal physical exhaustion, intensive care routine, difficulty of position and inadequate suction of the child on the nipple.^7^ ^,^ ^8^

Early BF is also seen as a challenge, due to the low rates of breastfeeding in the first hours of live around the world.^2^ The early beginning of breastfeeding represents a fundamental factor for its exclusive and prolonged continuity, not only due to the offer of colostrum and its benefits on the first postpartum days, but also due to the need of adaptation of the mother and the child in the process.^2^ The practice of BF stimulation during hospitalization requires the work of the health professionals involved, continuously and persistently. The availability of professionals trained to promote and support BF is an essential factor for this practice, especially in situations of difficulties.^6^ Therefore, it is important to mention the importance of establishing protective measures for BF involving all health professionals in the field to reduce early weaning.

It is observed that there are several recommendations for the stimulation of BF; however, in practice, the application is random. Despite the existing global policy of BF encouragement, called Baby-friendly Hospital Initiative (BFHI), many institutions, mainly the private ones, do not adhere to the program because of the autonomy of institutional routine and management of health indicators. However, regardless of obtaining the BFHI title, the practice of BF stimulation is a unanimous recommendation for the adequate health care provided for the women and the children. The conduction of a set of measures can increment the EBF rates of newborns and prevent early weaning. 

Therefore, this study aimed at identifying the main recommendations found in systematic reviews related with the protective factors of intrahospital EBF.

## METHOD

Integrative review according to the six established stages: elaboration of a guiding question, description of inclusion and exclusion criteria in the articles, search in the databases, analysis of the obtained data, discussion and presentation of results.^9^ We chose this method aiming at searching support to propose a set of measures addressed to protecting intrahospital EBF, through the current recommendations. 

The guiding question was: What is the evidence identified in the literature related with protective factors for EBF in the intrahospital period?

 The inclusion criteria of the studies were: systematic reviews that specifically dealt with the guiding question of the study, published between January 2007 and November 2016. We excluded the ones that analyzed the breastfeeding of premature children and with orofacial malformation. The search for publications was conducted in the databases Cochrane Library (Cochrane), US National Library of Medline (PubMed/MedLine) and *Literatura Latino-Americana e do Caribe em Ciências da Saúde* (LILACS), with the descriptor in *Medical Subject Headings* (MeSH) *Breast Feeding* and the word *Breastfeeding*.

The Cochrane database had, based on the descriptor and on the word related, 82 and 100 reviews, respectively, whereas LILACS had five and three reviews. In PubMed, the result was 1,633 publications in the search for the word, once it presented more articles. After the exclusion of the studies in duplicity and of those that did not correspond to the propose theme, the sample was composed of eight systematic reviews (Table 1). After the sample was identified, there was a data analysis through the full reading of the reviews in search of intrahospital actions that could contribute with EBF.

**Table 1:** Systematic reviews selected for the study.

N.	Title of the systematic review	Authors	Year
1	Interventions for promoting the initiation of breastfeeding^10^	Lisa Dyson, Felicia M McCormick and Mary J Renfrew	2005
2	Support for healthy breastfeeding mothers with healthy term babies^11^	Mary J Renfrew, Felicia M McCormick, Angela Wade, Beverley Quinn and Therese Dowswell	2012
3	Early skin-to-skin contact for mothers and their healthy newborn infants^12^	Elizabeth R Moore, Gene C Anderson, Nils Bergman and Therese Dowswell	2012
4	Breastfeeding promotion interventions and breastfeeding practices: a systematic review^13^	Sarah Haroon, Jai K Das, Rehana A Salam, Aamer Imdad, Zulfiqar A Bhutta	2013
5	Baby-led compared with scheduled (or mixed) breastfeeding for successful breastfeeding^14^	Anne Fallon, Deirdre Van der Putten, Cindy Dring, Edina H Moylett, Gerard Fealy and Declan Devane	2014
6	Interventions for treating painful nipples among breastfeeding women^15^	Cindy-Lee Dennis, Kim Jackson and Jo Watson	2014
7	Early additional food and fluids for healthy breastfed full-term infants^16^	Hazel A Smith and Genevieve E Becker	2016
8	Rooming-in for new mother and infant versus separate care for increasing the duration of breastfeeding^17^	Sharifah Halimah Jaafar, Jacqueline J Ho and Kim Seng Lee	2016

## RESULTS

The recommendations related with the protective factors of intrahospital EBF found in the systematic reviews were: early skin-to-skin contact; rooming-in care; intervention for treating painful nipples during breastfeeding; restriction of infant supplementation; baby-led breastfeeding; and educational interventions and individual or group support for mothers during hospital stay. The results of the systematic reviews are presented in Table 2.

**Table 2:** Summary of the conclusions related with the protective factors of breastfeeding in the reviews of the study.

N.	Objective of the systematic review	Conclusion of the systematic review
1	To assess the effectiveness of educational interventions on the BF initiation rate.	The educational interventions and the support to the mother on the first days of life increased the rates of BF initiation in comparison to routine care.
2	To assess the effectiveness of women’s care during breastfeeding.	The individual and/or group support incremented the duration of EBF in 4 to 6 weeks and in 6 months, respectively.
3	To assess the effect of early skin-to-skin contact on breastfeeding.	The early skin-to-skin contact presented a positive effect on the BF rate 1 to 4 months after birth, with increasing duration in 42 days. However, the exact time of skin-to-skin contact, the time and the technique used are not well established.
4	To identify the effect of educational interventions on BF rates up to 48 hours after delivery, on the 1st month and between 1 and 5 months.	The educational interventions significantly increased the rates of BF at birth in 43%; in the 1st month, in 30% and between the 1st and the 5th months, in 90%. The individual intervention increased the BF rates in 60%.
5	To assess baby-led breastfeeding in comparison to programmed breastfeeding in the success of the process.	Baby-led breastfeeding should be stimulated, according to current recommendations. However, there is no evidence to assess the effect of baby-led breastfeeding versus the controlled process.
6	To assess the effect of interventions to relieve mammillary pain and their impact on the duration and exclusivity of BF.	Human milk was equally beneficial in short-term mammillary pain in relation to another intervention with lanolin. The impact of the intervention on the duration and exclusivity of BF was not identified by the low quality of the studies to obtain this information.
7	To assess the benefits and negative aspects of additional foods or liquids for healthy at term children being breastfed and to determine the schedule and type of supplementation.	There was no difference between the use of artificial milk in the rates of BF at the time of hospital discharge, even though the children presented with lower rates of EBF at the age of 3 months. The evidence of benefits and possible negative effects of supplementation in children on the duration of BF was limited by the low quality of the studies.
8	To evaluate the effect of rooming-in care versus the separation of mother and child on the duration of BF.	The EBF rate on the 4th postpartum day, before hospital discharge, was significantly higher among the rooming-in care group.

BF: breastfeeding; EBF: exclusive breastfeeding.

## DISCUSSION

The reviews found showed the existing evidence of women and newborn care practices in the breastfeeding process, on the first days after delivery. The recommendations allowed identifying six actions that contribute with EBF in the intrahospital period.

### Early skin-to-skin contact

The delivery assistance hospital routine, for many years, prioritized the first care of the newborn, including actions such as using a warm crib and wrapping the child in a sterilized field, after birth. However, the contact with the mother, immediately after birth, has been recommended by the United Nations Children’s Fund (UNICEF),^18^ based on several short, mid and long-term benefits that the practice can provide, both for the child and the mother.^2^

Immediately after birth, the newborn goes through a moment of alert inactivity, which may last for 40 minutes. During this period, the direct contact with the mother’s skin not only develops a bond and provides favorable levels of heartbeat and breathing in the child, but also facilitates early breastfeeding and reduces the crying.^19^

Concerning its effect on BF, a systematic review verified the beneficial and adverse effects of early skin-to-skin contact on the exclusivity and duration of lactation, whose sample was of 34 studies involving a total of 2,177 mother-child dyads.^12^ The systematic review concluded that the early contact between mother and child was associated with EBF on the third postpartum day, and between one and four months after delivery, despite the great heterogeneity of the results and the varied methodological quality.^12^

### Rooming-in care

Rooming-in care is predicted and mandatory according to the Statute of the Child and the Adolescent (ECA).^20^ It is a hospitalization system in which the healthy newborn remains side by side with the mother 24 hours a day, right after birth until hospital discharge.^21^

The practice is considered as a facilitating measure for the beginning of BF, and ruled by Ordinance MS/GM n. 1.016/2003, which requires hospitals and maternity wards related with the Unifeid Health System (SUS) to implement this system. Also defended by BFHI, it is based on the advantages of stimulating baby-led breastfeeding, favoring the bond between mother and child through the early and continuous relationship, preventing hospital infection and enabling the mother to care for her child, which allows recognizing the needs of the child and clarifying doubts with the health professionals.^22^

The systematic review conducted in 2016 identified 26 trials with the purpose of assessing the duration of any BF and time of exclusive breastfeeding related with the practice of rooming-in care. However, only one randomized clinical study with 176 women and their respective children was included. This clinical trial compared the condition of the hospitalization of mother and baby in four groups: skin-to-skin contact right after birth followed by rooming-in care; no skin-to-skin contact at birth followed by rooming-in care; no skin-to-skin care at birth followed by nursery in the first 24 hours of life; and no skin-to-skin contact at birth followed by rooming-in care delayed up to three hours after birth. In the groups in which care was conducted separately, the breastfeeding took place seven times a day. In the rooming-in care groups, the choice was for baby-led breastfeeding.^17^ The EBF rate on the fourth postpartum day, before hospital discharge, was higher in the rooming-in care group, even though the results did not show any difference between the groups as to the duration of any BF until the fourth month.^17^

Despite the limited number of randomized clinical trials to assess the effect of the rooming-in care during breastfeeding, there are many benefits and no reasons not to do it. The exposure of the child to early and prolonged contact on the first days of life may provide a learning environment, enabling the establishment of BF and reducing the risk of weaning.^22^

### Intervention for treating painfulk nipples during breastfeeding

The report of pain during breastfeeding represents a major challenge for the health professional because it appears early, in the first two postpartum weeks, being one of the main causes of early weaning.^7^ ^,^ ^17^ Despite being frequent, many women believe it is a normal problem, which makes the situation worse, until she decides to ask for help.^23^

At first the women may present a mild discomfort in the region, on the first days of exposure of the nipples to the child’s sucking, but there is moderate to intense pain in 36 to 96% of the women who breastfeed,^24^ and the presence of mammillary lesion is the main cause of acute pain.^19^ There are many suggested treatments, besides correcting the cause, such as the inadequate sucking and position of the child.

The impact of nipple pain on the duration and exclusivity of BF was analyzed by a systematic review conducted in 2014, which assessed the existing interventions for handling nipple pain and its relationship with breastfeeding. The review included four clinical trials, contemplating 656 women and a total of five different types of interventions for nipple pain and/or nipple damage: glycerin protection, breast shells, only lanolin, only maternal milk and ointment in general (i.e., antifungal and antibacterial). All studies included guidance as to the correction of sucking and position of the child on the nipple and routine care.^15^

The application of maternal milk on the nipples was beneficial on the first postpartum days to prevent nipple pain before the seventh and the tenth days, once, after this period, the level of pain reported by the women was mild. There was not enough scientific evidence regarding the use of the other existing treatments for the improvement of the perception of nipple pain in women, when used alone or in association. As to the duration and exclusivity of BF, there was no difference between the types of breastfeeding proposed, considering the lack of quality in the existing clinical trials to identify this information.^15^

### Restricted use of supplementation 

Children’s supplementation or formula corresponds to the industrialized milk, which is mostly elaborated based on cow’s milk, recommended by the Brazilian Society of Pediatrics (SBP) only for children who cannot be breastfed.^25^ Even though there is a specific recommendation, its use is frequent, especially among mothers who have difficulties with breastfeeding.^26^ The restricted use of artificial milk and the non-offer of water and tea to children aged from 0 to months are recommendations from the World Health Organization (WHO) as a protective action to EBF.^1^

In the systematic review published in 2016, there were nine studies with 2,226 children aiming at, among other items, assessing the exclusivity of breastfeeding at the presence of additional foods or liquids in healthy at term children younger than six months of life.^16^

Regarding the use of artificial milk, there were no differences as to the use of complementation in the rates of BF at the time of hospital discharge, but the children presented lower rates of EBF at the age of three months.^16^

Concerning the use of water with glucose in the first 12 hours after birth, there was a reduction in episodes of hypoglycemia in comparison to EBF, however, without significant differences when applied in the next 12 hours. Such a supplementation brought no harm nor benefits to the duration of BF.^16^

The quality of the existing evidence about the use of formula and other liquids was insufficient to suggest any changes in the practice of EBF.^16^ The exposure of the child to other types of milk and/or liquids, besides the human milk, may reduce the benefits ensured by EBF, besides interfering in the sucking pattern of the child with the introduction of other feeding methods, such as the bottle, considered as a risk factor for weaning.^2^ ^,^ ^26^

### Baby-led breastfeeding

The baby-led breastfeeding refers to the time and duration of the breastfeeding being determined by the needs of the child. This practice has some benefits: it meets the needs of the child according to the demand, provides faster weight gain, leads to less neonatal jaundice, less crying, more stimulation of the breast and prevention of mammary ingurgitation, and causes the mother to learn how to respond to the child.^18^

The systematic review that assessed the effects of baby-led breastfeeding in comparison to the programmed or mixed breastfeeding, on the success of breastfeeding for healthy newborns, did not find randomized clinical trials to respond to the objects proposed.^14^ The authors state it is necessary to be more clear to define baby-led breastfeeding, and the divergence of this concept between both studies in the review made it impossible to compare the results. As a recommendation, they suggested that no changes should be made in the current guidelines without the identification of new evidence.^14^

### Educational interventions: individual and/or group support

Education is an important and complex instrument used by the health professional towards care, and this tool leads to a closer connection with the patients and the promotion of the quality of life in the population.^27^ The promotion and the support for the woman who breastfeeds play an essential role in the success of breastfeeding.^6^ ^,^ ^21^In this theme, we found three systematic reviews that approached the impact of educational interventions on BF and the support to the women in individual and/or group actions.

Concerning the educational intervention in health and its efficacy on BF, the systematic review conducted in 2005 analyzed the effect on rates of breastfeeding initiation.^10^ After the inclusion of five studies, with a total of 582 women in the postpartum, there was a significant increase in the number of women who initiated breastfeeding after the intervention. The educational intercession included group talks conducted daily in the hospital environment by the lactation consultant, telephone contact 48 hours after discharge, besides the schedule of a visit to the BF clinic with this professional one week after hospital discharge until weaning, or when the child completed one year of life.^10^

As to the individual and/or group support interventions, the systematic review conducted in 2012 found 52 randomized controlled studies in 21 countries, which included 56,451 binominals.^11^ Of these studies, 22 were conducted both in hospital postpartum nurseries and in community clinics: five studies in hospitals entitled Baby-Friendly; 30 only in community environments, and another study in five hospitals (two adapted to the BFHI rules and three with the certification).^11^The forms of intervention mentioned in this study included from additional training, individual and group support conducted in the hospital or in the community, until the telephone contact for breastfeeding support.^11^ In general, 36 of the 52 studies reported that people who provided care to breastfeeding had additional training to provide that kind of support to the women. In three studies, the professionals were certified at an international level by the International Board Certified Lactation Consultant (IBCLC).^11^ The group support, conducted both by laymen and health professionals, trained or not, was the most used in the studies and demonstrated a positive impact on the results of breastfeeding. All forms of support analyzed showed a significant effect on the early breastfeeding rates, on the increasing duration and exclusivity, proving the need for this support during the entire process of pregnancy and postpartum. The women who received in-person support presented about 20% less chances of abandoning EBF.^11^

The review published in 2013 also checked for the types of BF counselling (individual and in group) aiming at verifying its effect on the BF rates up to 48 hours after delivery, in the first month and between 1 and 5 months.^13^ The intervention in general demonstrated a significant effect, with an increase of 43% in EBF p to 48 hours after delivery. By comparing the type of intervention, the review showed a 60% increase between women who received individual support.^13^

### Set of the analyzed practices

By analyzing all recommendations related with the protective factors of intrahospital EBF found in the systematic reviews - object of this study -, we identified that five out of the six practices are part of the global BFHI strategy, which establishes, among other protective actions, the “Ten steps for the success of breastfeeding”. Launched by UNICEF and by the WHO in the early 1990s, it is part of a strategy conceived to respond to the call to action made by the Innocenti Declaration, which focuses on the need to increase the breastfeeding rates.^18^

The BFHI in Brazil began in March, 1992, with an action from the National Program of Breastfeeding Encouragement (PNIAM) and the Group of Defense of the Children’s Health, supported by the UNICED and the Pan American Health Organization (PAHO). After this period, a study showed a significant impact on the BF rates among children born in a registered hospital. The study showed that the time of EBF in the children born with the title versus those born without the title was, respectively, 60.2 and 48.1 days. The chances of breastfeeding in the first hour increased in 9% for those who were born in IHAC.^21^ Nowadays there are 323 institutions registered in Brazil, that is, 10% of the hospitals in Brazil.^28^ Even though the results have been significant, many institutions are probably not engaged, both for the difficulty to meet the demands to register in BFHI^22^ ^,^ ^28^ and because this is a public policy that involves interest and benefits only for those who provide services by SUS.^22^

However, it is believed that the acquisition of the BFHI title should be encouraged. The practices based on evidence regarding the women and children’s care is beyond the policy that encourages BF, and can be implemented by all hospital and maternity wards. The evidence found shows that the six practices of intrahospital BF encouragement are effective and increment the EBF rates, even though some studies had a methodological limitation.

## CONCLUSION

The early skin-to-skin contact, the rooming-in care, the intervention in mammillary pain during breastfeeding, the restricted use of supplementation, the baby-led breastfeeding and the individual and/or group educational interventions during hospitalization were the recommendations found, and they configure the proposal of the set of measures for the encouragement of intrahospital EBF.
